# The Effect of a Loading Dose Regimen in the Switch to Brolucizumab for Patients with Aflibercept-Resistant nAMD

**DOI:** 10.1155/2024/3673930

**Published:** 2024-01-30

**Authors:** Hiroyuki Kamao, Erika Mitsui, Yuto Date, Katsutoshi Goto, Kenichi Mizukawa, Atsushi Miki

**Affiliations:** ^1^Department of Ophthalmology, Kawasaki Medical School, 577 Matsushima, Kurashiki, Okayama 701-0114, Japan; ^2^Shirai Eye Hospital, 1339 Takasecho Kamitakase, Mitoyo, Kagawa 767-0001, Japan

## Abstract

**Purpose:**

To evaluate the one-year outcomes of switching to brolucizumab with and without a loading dose regimen (three monthly injections) in eyes with aflibercept-resistant neovascular age-related macular degeneration (nAMD).

**Methods:**

We retrospectively studied nAMD patients who had retinal exudate under bimonthly injections of aflibercept and were switched to brolucizumab from aflibercept. Patients were grouped into intravitreal brolucizumab injection (IVBr) with a loading dose regimen (loading group) and without a loading dose regimen (nonloading group). We assessed the best-corrected visual acuity (BCVA), central retinal thickness (CRT) at the fovea, subfoveal choroidal thickness (SFCT), IVBr status (number of injections and last injection interval), and retinal exudate status on optical coherence tomography.

**Results:**

Overall, 52 eyes received ≥1 IVBr; 26 eyes received ≥3 IVBr with 12-month follow-up. A total of 13 eyes in the loading group and 13 eyes in the nonloading group were reviewed. One year after switching, BCVA changed from 0.28 ± 0.25 to 0.19 ± 0.28 in the loading group (*P*=0.28) and from 0.25 ± 0.20 to 0.23 ± 0.25 in the nonloading group (*P*=0.92). The mean CRT decreased from 263.6 ± 40.7 *µ*m to 221.7 ± 54.6 *µ*m in the loading group (*P*=0.03), while it only changed from 244.9 ± 77.2 *µ*m to 221.0 ± 78.7 *µ*m in the nonloading group (*P*=0.26). Both the loading and nonloading groups achieved 69% dry macula. The number of injections received was significantly higher in the loading group (7.6 ± 0.6 vs. 6.8 ± 0.4, *P*  <  0.001). Two patients (4.2%) developed intraocular inflammation.

**Conclusion:**

Switching to brolucizumab from aflibercept for eyes with nAMD with resistance to bimonthly injections of aflibercept is a valuable treatment option with and without the loading regimen. This trial is registered with UMIN000023676.

## 1. Introduction

Neovascular age-related macular degeneration (nAMD) is a leading cause of blindness in elderly populations in developed countries [1]. Antivascular endothelial growth factor (VEGF) agents have improved visual outcomes in patients with nAMD [2]. However, the requirement of long-term administration of expensive anti-VEGF agents causes poor medication adherence [3], and undertreatment represents a risk of visual deterioration [4]. Hence, longer-lasting anti-VEGF agents are needed.

Brolucizumab is an anti-VEGF agent of a humanized single-chain antibody fragment approved for patients with nAMD in 2019 [5]. Its molecular weight of 26 kDa, the smallest molecular weight among commercially available anti-VEGF agents, allows it to be given in higher molar doses. In two prospective randomized phase 3 trials (HAWK and HARRIER) of brolucizumab for treatment-naïve nAMD patients, brolucizumab achieved noninferior visual outcomes and superior anatomical outcomes compared to aflibercept [6, 7]. Therefore, brolucizumab has received attention as an anti-VEGF agent that improves medication adherence. Aflibercept [8], widely used as an anti-VEGF agent for nAMD, is usually administered at 8-16-week intervals [9], though approximately 30% of nAMD patients treated with aflibercept require 4- or 6-week treatment intervals [10, 11]. Recent studies have shown the efficacy of switching to intravitreal injection of brolucizumab (IVBr) for patients with nAMD refractory to aflibercept [12, 13]. When nAMD patients switch anti-VEGF agents, three monthly intravitreal injections as a loading dose regimen are recommended to start, though the administration interval that had been used before switching is sometimes continued in clinical practice. The effectiveness of the loading dose regimen in such cases is unknown. Herein, we compared the 1-year visual outcomes and anatomical responses of patients with nAMD refractory to aflibercept who were switched from aflibercept to brolucizumab with a loading dose regimen (loading group) vs. those who were switched from aflibercept to brolucizumab without a loading dose regimen (nonloading group).

## 2. Materials and Methods

We performed this study, which was conducted according to the Declaration of Helsinki principles and registered with the UMIN Clinical Trials Registry (UMIN000023676), with approval from the Kawasaki Medical School Ethics Committee (2543-03 and 5954-00). We obtained informed consent from patients via an opt-out method through the website of Kawasaki Medical School. We retrospectively studied 52 eyes of 49 consecutive nAMD patients who had retinal exudate, including intraretinal fluid (IRF) and subretinal fluid (SRF), under intravitreal aflibercept injection and were switched to brolucizumab from aflibercept at Kawasaki Medical School between July 2020 and April 2023. Upon the decision to switch to IVBr, one physician (H.K.) proposed the loading dose regimen (three initial monthly injections) to all patients. If consent was not secured, patients underwent brolucizumab administration without adherence to the loading dose regimen. Twenty-seven eyes of 27 nAMD patients consented to the loading dose regimen. We excluded 26 eyes of 23 patients who were followed up for less than one year after the medications were switched. All patients were treated with the treat-and-extend protocol of IVBr, which were adjusted for 2 to 4 weeks based on the retinal exudate findings (IRF and SRF) on OCT. The treatment interval was extended when no retinal exudate occurred for six months and reduced when the retinal exudate remained. As the primary outcome, the proportion of dry macula at the last visit was compared between the loading and nonloading groups. Data regarding cigarette smoking, hypertension, and diabetes were collected from patient recall or hospital records. We divided the patients into never-smokers and ever-smokers as described in a previous report [14]. All participants received a complete ophthalmologic examination, including measurement of best-corrected visual acuity (BCVA), indirect ophthalmoscopy, slit-lamp biomicroscopy with a noncontact lens, color fundus photography and fundus autofluorescence (Canon CX-1; Canon, Tokyo, Japan), swept-source optical coherence tomography (OCT) (DRI OCT-1 Atlantis; Topcon Corporation, Tokyo, Japan), and fluorescein and indocyanine green angiography (HRA-2; Heidelberg Engineering GmbH, Dossenheim, Germany). Visual acuity data were obtained in decimal BCVA and converted to the logarithm of the minimum angle of resolution (logMAR) units for the analysis. The retinal and choroidal thicknesses were measured by swept-source OCT as described in a previous report [15].

The statistical analyses were performed by JMP Pro 17 software (SAS Institute). Age, follow-up period before switching, number of anti-VEGF therapies before switching, best-corrected visual acuity (BCVA), central retinal thickness (CRT), subfoveal choroidal thickness (SFCT), number of brolucizumab injections received, and brolucizumab injection interval were compared between the nAMD patients switched with and without the loading regimen by the Wilcoxon rank-sum test. The chi-square test was used to compare the differences in proportions of females, hypertension, diabetes, ever-smokers, proportions of AMD subtypes, history of receiving PDT, and dry macula between the two groups. Steel's multiple comparison test was used to compare the differences in BCVA, CRT, and SFCT changes at 0, 6, and 12 months. *P* values < 0.05 were considered statistically significant in all analyses (^*∗*^*P* < 0.05; ^*∗∗*^*P* < 0.01; ^†^*P* < 0.001).

## 3. Results

In total, 26 eyes of 26 patients with nAMD switched from aflibercept to brolucizumab were assessed at the one-year follow-up evaluation. The baseline characteristics of the nAMD patients before switching are presented in Table 1. The analysis included 13 patients (3 women and 10 men; mean [±SD] age 77.2 ± 7.2 [range, 65–92] years) in the loading group and 13 patients (4 women and 9 men; mean age 79.1 ± 7.3 [range, 69–91] years) in the nonloading group. The proportions of hypertension, diabetes, and ever-smoker status were 38.5%, 23.1%, and 61.5% in the loading group and 30.8%, 15.4%, and 76.9% in the nonloading group, respectively. The proportions of typical AMD (type 1 and type 2 macular neovascularization), polypoidal choroidal vasculopathy (PCV), and retinal angiomatous proliferation (RAP, also called type 3 macular neovascularization) were 23.1%, 76.9%, and 0.0% in the loading group and 30.8%, 61.5%, and 7.7% in the nonloading group, respectively. The mean follow-up period before switching from aflibercept to brolucizumab was 40.9 ± 22.2 months in the loading group and 56.4 ± 49.0 months in the nonloading group. The mean number of anti-VEGF therapies before switching from aflibercept to brolucizumab was 29.1 ± 21.0 in the loading group and 28.7 ± 25.8 in the nonloading group. Four patients received photodynamic therapy (PDT) in the loading group, and three patients in the nonloading group. The two study groups were comparable in age, female-to-male ratio, proportions of hypertension, diabetes, ever-smokers, nAMD subtypes, history of receiving PDT, follow-up period, and number of anti-VEGF therapies before switching.

**Figure 1:**
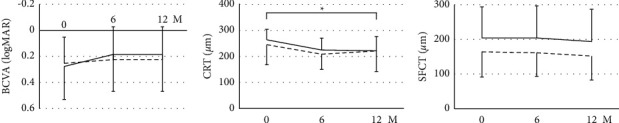
Changes in the mean BCVA, CRT, and SFCT from before to after switching to brolucizumab.

**Figure 2:**
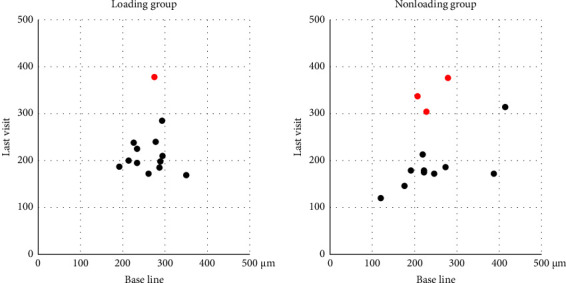
Scatterplot of the central retinal thickness before and after switching to brolucizumab. The red dots represent eyes with a ≥50 *μ*m increase in retinal thickness at 12 months compared to Base line.

Table 2 and Figure 1 summarize the one-year treatment outcomes of the two groups. The mean BCVA at baseline and at the final visit was 0.28 ± 0.25 and 0.19 ± 0.28 in the loading group and 0.25 ± 0.20 and 0.23 ± 0.25 in the nonloading group, respectively; the groups were similar at both times (baseline: *P*=0.98; final visit: *P*=0.59). There were no significant differences in BCVA changes at 0, 6, and 12 months in either group. The mean CRT at baseline and at the final visit was 263.6 ± 40.7 *µ*m and 221.7 ± 54.6 *µ*m in the loading group and 244.9 ± 77.2 *µ*m and 221.0 ± 78.7 *µ*m in the nonloading group, respectively, which were also similar at both times (baseline: *P*=0.14; final visit: *P*=0.41). The mean CRT decreased from baseline to the final visit in the loading group (*P*=0.03), but it did not in the nonloading group (*P*=0.13). One eye in the loading group and three eyes in the nonloading group had a retinal thickness that increased by 50 *μ*m or more over the 12 months after the switch (Figure 2). The mean SFCT at baseline and at the final visit was 203.7 ± 89.8 *µ*m and 193.5 ± 93.7 *µ*m in the loading group and 164.1 ± 73.0 *µ*m and 151.8 ± 69.3 *µ*m in the nonloading group, respectively; the groups were similar at both times (baseline: *P*=0.28; final visit: *P*=0.20). There was no significant change in SFCT at 0, 6, and 12 months in either group.

The solid line represents the loading group, and the dashed line represents the nonloading group.

At 12 months, 9 out of 14 eyes (69.2%) in each group achieved a dry macula (*P*=1.00).

The mean number of injections received in the loading and nonloading groups was 7.6 ± 0.6 per year and 6.8 ± 0.4 per year, respectively; the number of injections received was significantly higher in the loading group (*P*=0.001). At 12 months, the injection interval in the loading group was 8 weeks in 11 eyes (84.6%) and 10 weeks in 2 eyes (15.4%), for the mean injection interval of 8.3 ± 0.7 weeks. The injection interval in the nonloading group was 8 weeks in 8 eyes (61.5%), 10 weeks in 3 eyes (23.1%), and 12 weeks in 2 eyes (15.4%), for the mean injection interval of 9.1 ± 1.5 weeks. The proportion of injection interval and mean injection interval were similar (proportion of injection interval: *P*=0.09; injection interval: *P*=0.17). Four eyes in each group had SRF at 12 months, and all 8 eyes were treated with bimonthly injections of brolucizumab.

One eye of one patient in each group dropped out due to the development of brolucizumab-associated IOI and retinal vasculitis after the first injection. Both patients were female and were treated with steroid therapy (0.1% betamethasone eye drops four times daily and subtenon triamcinolone acetonide injection), and the treatment was switched back to aflibercept after resolving their inflammation.

## 4. Discussion

The present study revealed that switching from aflibercept to brolucizumab with and without the loading dose regimen was effective in controlling retinal exudate in nAMD patients who had IRF and/or SRF under aflibercept administration at 8-week intervals. These results are consistent with previous findings in anti-VEGF treatment-resistant nAMD patients [12, 13]. Kikushima et al. reported the one-year results of switching to brolucizumab in nAMD patients who had retinal fluid after 4–8 weeks of aflibercept administration. The switch to brolucizumab achieved a dry macula in 43% of the eyes and extended the mean injection interval by 4.5 weeks. Haensli et al. showed that 29% of the eyes achieved dry macula, and the mean injection interval was extended for 3.7 weeks in nAMD patients with persisting retinal fluid under 4–6 week intervals of aflibercept or ranibizumab administration. In this study, the proportion of dry macula and extension of the injection interval was 69% and 0.3 weeks in the loading group and 69% and 1.1 weeks in the nonloading group. We found a higher proportion of dry macula and a lesser extension of the injection interval than previous studies. In the extension of the injection interval by switching to brolucizumab, previous reports demonstrated that nAMD patients treated with a shorter injection interval before switching showed a longer extension of the injection interval [16, 17]. Coney et al. reported that eyes with preswitch injection intervals <8 weeks showed a 23.6-day longer extension of the injection interval than those with preswitch injection intervals ≥8 weeks. MacCumber et al. reported that the extenders who had extended their injection intervals by two weeks or longer at 12 months were the ones who had significantly shorter preswitch injection intervals than nonextenders (preswitch injection interval: extenders 5.9 weeks and nonextenders 10.1 weeks). Differences in our results from other studies' results could be caused by the difference in the preswitch injection intervals.

This study revealed that the nonloading group achieved the same dry macular rate and 0.8 fewer injections per year compared to the loading group. The cost-effectiveness analysis of anti-VEGF drugs in various clinical studies, such as the MARINA [18] and ANCHOR [19] trials, led to the initiation of anti-VEGF treatment with three monthly intravitreal injections as the loading dose regimen. However, few studies have examined the efficacy of loading dose regimens for treatment-naïve nAMD patients [20], and the effectiveness of the loading dose regimen in switching to another anti-VEGF drug is unknown. The reduction in the number of injections improves medication adherence; therefore, our results indicate that switching to brolucizumab without a loading dose regimen is a valuable treatment option for patients with aflibercept-resistant nAMD.

The mean CRT significantly decreased in the loading group but nonsignificantly decreased in the nonloading group. This difference could have been caused by the higher number of patients with a 50 *µ*m or more increase in retinal thickness from baseline to 12 months in the nonloading group. In various diseases, initial treatment often involves higher doses of drugs to rapidly control disease activity. Our results suggest that some patients need the loading doses regimen to control the retinal exudate even if switching to another anti-VEGF drug. However, it remains unclear which patients need the loading dose regimen, and further studies are needed to elucidate markers for identifying patients requiring the loading dose regimen.

In the present study, the loading and nonloading groups had no visual improvement despite anatomical improvement. Improvements in anatomical outcomes without visual improvement have been reported in switching to another anti-VEGF drug [12, 13, 21]. We speculate that this discrepancy was due to permanent damage to photoreceptors caused by long-term disease activity and treatment.

IOI is a clinical problem that has received attention since the approval of brolucizumab for nAMD by the U.S. Food and Drug Administration in October 2019. This brolucizumab-associated IOI ranges from mild iritis without symptoms to irreversible visual disturbance due to retinal vascular occlusion. The HAWK/HARRIER study reported that the incidence of brolucizumab-associated IOI was 4.6% for iritis/vitritis, 3.3% for retinal vasculitis, and 2.1% for retinal vascular occlusion [22]. A systematic review of brolucizumab-associated IOI showed that most patients were female (almost 80%), and approximately 46% of eyes had onset after the first brolucizumab injection [23]. In this study, the incidence of IOI was 3.8% (2/52), and both patients were female. Patients with nAMD in Japan are more prevalent in males, and females only made up 34.7% (17/49) of our sample; hence, the administration of brolucizumab to females may require close follow-up.

The limitations of this study include its retrospective nature and its relatively small sample. This study has a selection bias, as a single physician proposed the loading dose regimen to all patients at the switching to brolucizumab. While no substantial differences in patient characteristics were observed between the two groups, the follow-up period prior to the switching and retinal thickness was relatively longer and lower in the nonloading group compared to the loading group, respectively. Prolonged administration of anti-VEGF agents could reduce the exudative changes as disease activity. In addition, prolonged treatment causes poor medication adherence, so patients may have requested switching without the loading dose regimen. Prolonged treatment causes anatomical changes that may influence the effect of switching to brolucizumab; therefore, the effect of the loading regimen requires validation by larger prospective studies.

This is the first report to demonstrate the effect of a loading dose regimen in the switching from aflibercept to brolucizumab. All of our subjects were nAMD patients who had retinal exudate when they were receiving aflibercept at 8-week intervals.

## 5. Conclusions

Both the loading and nonloading groups achieved 69% dry macula; therefore, switching to brolucizumab from aflibercept for eyes with nAMD with resistance to bimonthly injections of aflibercept is a valuable treatment option with and without the loading regimen.

## Figures and Tables

**Table 1:** Clinical characteristics of nAMD patients who switched to brolucizumab with and without the loading dose regimen.

	Loading group (*n* = 13)	Nonloading group (*n* = 13)	*P*
Age (years), mean (SD)	77.2 (7.2)	79.1 (7.3)	0.55
Sex (female), no. (%)	3 (23.1)	4 (30.8)	0.66
Hypertension, no. (%)	5 (38.5)	4 (30.8)	0.68
Diabetes, no. (%)	3 (23.1)	2 (15.4)	0.62
Smoking habits (ever-smokers), no. (%)	8 (61.5)	10 (76.9)	0.39
AMD subtype, no. (%)			0.42
Typical AMD	3 (23.1)	4 (30.8)	
PCV	10 (76.9)	8 (61.5)	
RAP	0 (0.0)	1 (7.7)	
Follow-up period (mo.), mean (SD)	40.9 (22.2)	56.4 (49.0)	0.98
Total injections (no.), mean (SD)	29.1 (21.0)	28.7 (25.8)	0.84
History of receiving PDT, no. (%)	4 (30.8)	3 (23.1)	0.66

**Table 2:** One-year outcomes of switching to brolucizumab with and without the loading dose regimen in nAMD patients.

	Loading group (*n* = 13)	Nonloading group (*n* = 13)	*P*
Baseline, mean (SD)			
BCVA (logMAR)	0.28 (0.25)	0.25 (0.20)	0.98
CRT (*µ*m)	263.6 (40.7)	244.9 (77.2)	0.14
SFCT (*µ*m)	203.7 (89.8)	164.1 (73.0)	0.28
Outcome, mean (SD)			
BCVA (logMAR)	0.19 (0.28)	0.23 (0.25)	0.59
CRT (*µ*m)	221.7 (54.6)	221.0 (78.7)	0.41
SFCT (*µ*m)	193.5 (93.7)	151.8 (69.3)	0.2
Change, mean (SD)			
BCVA (logMAR)	−0.09 (0.14)	−0.03 (0.13)	0.32
CRT (*µ*m)	−41.9 (66.6)	−23.9 (87.3)	0.64
SFCT (*µ*m)	−10.2 (21.6)	−12.2 (11.4)	0.74
Dry macular, number (%)	9 (69.2)	9 (69.2)	1.00
Number of injections, mean (SD)	7.6 (0.6)	6.8 (0.4)	0.001^†^
Injection interval (week), mean (SD)	9.2 (2.5)	9.5 (2.4)	0.51

## Data Availability

The data used to support the findings of this study are restricted by the Kawasaki Medical School Ethics Committee to protect patient privacy. Data are available from Hiroyuki, Kamao MD, Ph.D. [hironeri@med.kawasaki-m.ac.jp] for researchers who meet the criteria for access to confidential data.

## References

[1] Bressler N. M., Bressler S. B., Fine S. L. (1988). Age-related macular degeneration. *Survey of Ophthalmology*.

[2] Rosenfeld P. J., Brown D. M., Heier J. S. (2006). Ranibizumab for neovascular age-related macular degeneration. *New England Journal of Medicine*.

[3] Boulanger-Scemama E., Querques G., About F. (2015). Ranibizumab for exudative age-related macular degeneration: a five year study of adherence to follow-up in a real-life setting. *Journal Français d’Ophtalmologie*.

[4] Boyer D. S., Heier J. S., Brown D. M., Francom S. F., Ianchulev T., Rubio R. G. (2009). A phase IIIb study to evaluate the safety of ranibizumab in subjects with neovascular age-related macular degeneration. *Ophthalmology*.

[5] Tadayoni R., Sararols L., Weissgerber G., Verma R., Clemens A., Holz F. G. (2021). Brolucizumab: a newly developed anti-VEGF molecule for the treatment of neovascular age-related macular degeneration. *Ophthalmologica*.

[6] Dugel P. U., Koh A., Ogura Y. (2020). HAWK and HARRIER: phase 3, multicenter, randomized, double-masked trials of brolucizumab for neovascular age-related macular degeneration. *Ophthalmology*.

[7] Dugel P. U., Singh R. P., Koh A. (2021). HAWK and HARRIER: ninety-six-week outcomes from the phase 3 trials of brolucizumab for neovascular age-related macular degeneration. *Ophthalmology*.

[8] Heier J. S., Brown D. M., Chong V. (2012). Intravitreal aflibercept (VEGF Trap-Eye) in wet age-related macular degeneration. *Ophthalmology*.

[9] Ohji M., Takahashi K., Okada A. A. (2020). Efficacy and safety of intravitreal aflibercept treat-and-extend regimens in exudative age-related macular degeneration: 52- and 96-week findings from ALTAIR. *Advances in Therapy*.

[10] DeCroos F. C., Reed D., Adam M. K. (2017). Treat-and-extend therapy using aflibercept for neovascular age-related macular degeneration: a prospective clinical trial. *American Journal of Ophthalmology*.

[11] MacCumber M. W., Yu J. S., Sagkriotis A. (2023). Antivascular endothelial growth factor agents for wet age-related macular degeneration: an IRIS registry analysis. *Canadian Journal of Ophthalmology*.

[12] Haensli C., Pfister I. B., Garweg J. G. (2021). Switching to brolucizumab in neovascular age-related macular degeneration incompletely responsive to ranibizumab or aflibercept: real-life 6 month outcomes. *Journal of Clinical Medicine*.

[13] Kikushima W., Sakurada Y., Fukuda Y. (2023). A treat-and-extend regimen of intravitreal brolucizumab for exudative age-related macular degeneration refractory to aflibercept: a 12-month result. *Pharmaceuticals*.

[14] Kamao H., Goto K., Mito Y., Miki A., Kiryu J. (2018). Effects of smoking on outcomes of antivascular endothelial growth factor therapy in patients with neovascular age-related macular degeneration smoking and anti-VEGF Therapy in nAMD. *Journal of Ophthalmology*.

[15] Kamao H., Goto K., Matsuno K., Mizukawa K., Miki A., Kiryu J. (2021). Clinical characteristics of neovascular age-related macular degeneration without typical drusen. *Journal of Ophthalmology*.

[16] MacCumber M. W., Wykoff C. C., Karcher H. (2023). Factors linked to injection interval extension in eyes with wet age-related macular degeneration switched to brolucizumab. *Ophthalmology*.

[17] Coney J. M., Zubricky R., Sinha S. B. (2023). Switching to brolucizumab: injection intervals and visual, anatomical and safety outcomes at 12 and 18 months in real-world eyes with neovascular age-related macular degeneration. *International Journal of Retina and Vitreous*.

[18] Boyer D. S., Antoszyk A. N., Awh C. C., Bhisitkul R. B., Shapiro H., Acharya N. R. (2007). Subgroup analysis of the MARINA study of ranibizumab in neovascular age-related macular degeneration. *Ophthalmology*.

[19] Brown D. M., Michels M., Kaiser P. K., Heier J. S., Sy J. P., Ianchulev T. (2009). Ranibizumab versus verteporfin photodynamic therapy for neovascular age-related macular degeneration: two-year results of the ANCHOR study. *Ophthalmology*.

[20] Gupta B., Adewoyin T., Patel S.-K., Sivaprasad S. (2011). Comparison of two intravitreal ranibizumab treatment schedules for neovascular age-related macular degeneration. *British Journal of Ophthalmology*.

[21] Lim R. H., Gupta B., Simcock P. (2017). Intravitreal aflibercept in neovascular age-related macular degeneration previously treated with ranibizumab. *International Journal of Ophthalmology*.

[22] Monés J., Srivastava S. K., Jaffe G. J. (2021). Risk of inflammation, retinal vasculitis, and retinal occlusion-related events with brolucizumab: post hoc review of HAWK and HARRIER. *Ophthalmology*.

[23] Wykoff C. C., Matsumoto H., Barakat M. (2023). Retinal vasculitis or vascular occlusion after brolucizumab for neovascular age-related macular degeneration. A systematic review of real-world evidence. *Retina*.

